# Synthesis, Characterization, and Electronic Properties of ZnO/ZnS Core/Shell Nanostructures Investigated Using a Multidisciplinary Approach

**DOI:** 10.3390/ma16010326

**Published:** 2022-12-29

**Authors:** Jelena Zagorac, Dejan Zagorac, Vesna Šrot, Marjan Ranđelović, Milan Pejić, Peter A. van Aken, Branko Matović, J. Christian Schön

**Affiliations:** 1 Materials Science Laboratory, “Vinča” Institute of Nuclear Sciences—National Institute of the Republic of Serbia, University of Belgrade, 11000 Belgrade, Serbia; 2 Centre of Excellence “Cextreme Lab”, Materials Science Laboratory, “Vinča” Institute of Nuclear Sciences—National Institute of the Republic of Serbia, University of Belgrade, 11000 Belgrade, Serbia; 3 Max Planck Institute for Solid State Research, Stuttgart Center for Electron Microscopy, 70569 Stuttgart, Germany; 4 Department of Chemistry, Faculty of Mathematics and Natural Sciences, University of Niš, 18000 Niš, Serbia; 5 Nanoscale Science Department, Max Planck Institute for Solid State Research, 70569 Stuttgart, Germany

**Keywords:** ZnO/ZnS, core/shell, band gap, XRD, HR-(S)TEM, ab initio

## Abstract

ZnO/ZnS core/shell nanostructures, which are studied for diverse possible applications, ranging from semiconductors, photovoltaics, and light-emitting diodes (LED), to solar cells, infrared detectors, and thermoelectrics, were synthesized and characterized by XRD, HR-(S)TEM, and analytical TEM (EDX and EELS). Moreover, band-gap measurements of the ZnO/ZnS core/shell nanostructures have been performed using UV/Vis DRS. The experimental results were combined with theoretical modeling of ZnO/ZnS (hetero)structures and band structure calculations for ZnO/ZnS systems, yielding more insights into the properties of the nanoparticles. The ab initio calculations were performed using hybrid PBE0 and HSE06 functionals. The synthesized and characterized ZnO/ZnS core/shell materials show a unique three-phase composition, where the ZnO phase is dominant in the core region and, interestingly, the auxiliary ZnS compound occurs in two phases as wurtzite and sphalerite in the shell region. Moreover, theoretical ab initio calculations show advanced semiconducting properties and possible band-gap tuning in such ZnO/ZnS structures.

## 1. Introduction

The availability of suitable nanomaterials is of great importance in the field of nanotechnology. These materials have wide industrial and ecological applications (magnets, batteries, catalysts, and electronics) [1,2,3,4,5,6,7,8]. In particular, materials that exhibit compositional variation on the nanoscopic level, such as heterolayers or core/shell nanostructures, e.g., oxysulfides synthesized via sulfidation in an anion exchange reaction, are of great interest [1,9,10,11,12,13,14,15,16,17]. In the literature, core/shell structures show upgraded optoelectronic, electrochemical, and magnetic properties in comparison with their pristine components [12,13,16,18,19]. One material where such core/shell nanostructures are expected to be of much promise is the ZnO/ZnS system. Its pristine components are zinc oxide (ZnO) and zinc sulfide (ZnS), which both can crystallize in the cubic sphalerite and the hexagonal wurtzite modification, depending on the synthesis conditions [20,21,22,23,24,25,26,27]. ZnO has been subject to extensive research activities due to its direct and wide band gap (~3.4 eV), and its nanostructures have been recently studied due to their unique electronic and optoelectronic properties [14,28,29,30,31,32,33], while ZnS, which possesses an even wider band gap (~3.7 eV), is a promising candidate for coating materials and has potential applications in electroluminescent devices, sensors, and lasers [34,35,36,37].

Despite their electronegativity and size differences, the electronic structure of ZnO and ZnS can be modified via the replacement of oxygen by sulfur in ZnO or sulfur by oxygen in ZnS [38,39,40]. Zinc oxysulfide was first reported 30 years ago as thin films grown by atomic layer deposition (ALD) [39]. In the last decade, extensive syntheses and characterizations of ZnO/ZnS thin films were reported, involving ALD [41], pulsed laser deposition [42], and chemical spray pyrolysis [43]. Moreover, ZnO/ZnS heterostructures and heterojunctions with various morphologies have been recently reported [14,44,45,46,47,48], including core/shell nanostructures which are the topic of this investigation.

The first studies on ZnO/ZnS solid solutions took place in 1960 [49], and later, various research groups studied small non-stoichiometric effects (e.g., doping, vacancies, etc.) on either ZnS or ZnO, causing improvements in semiconducting and optical properties, and leading to the deduction of structure–property relationships [50,51,52,53,54]. There are also rare reports of combined ZnO and ZnS mineral forms appearing in nature, e.g., where twin boundaries deficient in sulfur and enriched in oxygen have been observed [55,56].

Recently, ZnO/ZnS heterostructures with heteroepitaxy of single-crystalline ZnO/ZnS core/shell nanowire arrays have been realized using amorphous HfO_2_ as the buffer layer [57]. Many different methods have been used to synthesize ZnO/ZnS core–shell structures. These methods include, for example, hydrothermal fabrication on glass substrates [17], syntheses from an aqueous solution including precipitation [58], a one-step thermal evaporation method [59], a solid–vapor process [44], sulfuration of the ZnO template [60], thermal evaporation [61], and a wet chemical synthesis route [62]. In particular, the hydrothermal-supported co-precipitation method is one of the ways to synthesize ZnO/ZnS core–shell nanostructures [63].

Concerning the electronic properties, there are many earlier reports in the literature on theoretical and experimental band-gap values of wurtzite and sphalerite type ZnO_1−x_S_x_ compounds with different compositions (x) [18,38,40,64,65,66,67,68,69]. These ZnO/ZnS nanomaterials mostly exhibit core/shell structures and have found very wide technological applications such as solar cells, displays, optoelectronic devices, catalysis, biosensors, electronics, magnetism, mechanics, electrochemistry, nanostructures, semiconductors for photovoltaics, light-emitting diodes (LED), heterostructures, infrared detectors, thermoelectrics, etc. [15,59,70,71,72,73,74,75]. The present study provides detailed investigations of structural and electronic properties of ZnO/ZnS core/shell nanostructures, using a multidisciplinary approach [76,77] combining experimental methods such as XRD, HR-(S)TEM, and analytical TEM (EDX and EELS), and band-gap measurements, with ab initio structure optimizations and band structure calculations.

## 2. Materials and Methods

### 2.1. Experimental Details

#### 2.1.1. Synthesis

For the synthesis of ZnO/ZnS core–shell nanostructures, gas-phase sulfidation of ZnO powder (Merck, Darmstadt, Germany, high purity of 99.99%, diameter of the nanoparticles O (100 nm)) at elevated temperatures was employed. Hydrogen sulfide was initially obtained from a Kipp apparatus and, without further processing, was introduced in the round-bottom flask containing 3 g of ZnO powder. During the synthesis, the round-bottom flask was heated to 340–400 °C while H_2_S gas passed with a flow of 6.5 mL/min for 6 h. Vinyl laboratory tubings were used to introduce gas into the flow reactor. Regarding the reaction products at the end of synthesis, they were allowed to cool down to room temperature. After that, the solid phase was removed from the vessel and packed into tightly closed containers for further characterization. Reaction products are the core–shell ZnO/ZnS powder and the water vapor, which was removed with the flow of the H_2_S gas. Unreacted H_2_S was collected and retained in two stages. First, a vessel containing a FeCl_3_ solution was used to chemically convert H_2_S gas into iron sulfides, and the small amount of gas remaining was caught in the next stage using a solution of NaOH.

#### 2.1.2. Crystallographic Studies

The ZnO/ZnS core–shell particles obtained were characterized by X-ray powder diffraction (XRPD) with a Riguku Ultima IV diffractometer using Cu-Kα radiation and a Ni filter. To derive the relevant structural parameters, experimental data were taken at a speed of 2° per min in a range of diffraction angles 2θ (10–90°), with an angular resolution of 0.02°. Structural analysis was carried out using the ICSD database [78], Rietveld refinement, and the program FullProf [79].

#### 2.1.3. Electron Microscopy

Scanning electron microscopy (SEM) images were collected on a field-emission LEO microscope (Zeiss, Jena, Germany) at a working distance of 3–6 mm and with an accelerating voltage of 2–3 kV. A transfer tool between the SEM working chamber and a glovebox was used for inserting the samples (cycled electrodes) in order to avoid their exposure to air during transfer. High-resolution transmission electron microscopy (HRTEM), bright-field (BF), and high-angle annular dark-field (HAADF) scanning TEM (STEM) imaging were combined with energy-dispersive X-ray spectroscopy (EDX) with an advanced TEM (JEOL ARM200F, JEOL Co. Ltd., Tokyo, Japan), equipped with a cold field-emission gun and a CETCOR image corrector (CEOS GmbH, Heidelberg, Germany). EDX elemental maps were obtained by acquiring area scans using a JEOL JED-2300 DrySD^TM^ detector. EELS elemental maps (2D spectrum images) were obtained in STEM mode with a post-column energy filter with dual EELS acquisition capability (Gatan GIF Quantum ERS, Gatan Inc., Pleasanton, CA, USA) with a dispersion of 1 eV/channel.

#### 2.1.4. UV/Vis Diffuse-Reflectance Spectroscopy

Electronic band-gap measurements were performed using powder diffuse-reflectance spectroscopy (DRS) and Tauc plots for the synthesized ZnO/ZnS samples, which had been characterized by XRD, (S)TEM, and analytical TEM. Reflectance spectra were measured using undiluted powders of ZnO/ZnS core–shell particles. Diffuse powder reflectance spectra (4000 ≤ ṽ ≤ 35000 cm^−1^) were recorded at ambient temperature using two modified CARY 14 and CARY 17 spectrophotometers (OLIS, Inc., Athens, GA, USA), which were equipped with integrating spheres.

### 2.2. Computational Details

#### 2.2.1. Ab Initio Structure Optimization

The ab initio calculations were conducted using the CRYSTAL17 software package [80,81], based on linear combinations of atomic orbitals (LCAO). The ZnO/ZnS models had been generated using the Supercell method [82,83] and the primitive cell approach for atom exchange (PCAE) method [84,85]. The ab initio structure optimizations included analytical gradients [86] and were performed using two hybrid functionals: HSE06 and PBE0. The hybrid HSE06 (Heyd–Scuseria–Ernzerhof) exchange–correlation functional employs an error-function-screened Coulomb potential to compute the exchange portion of the energy to improve computational efficiency [87].The PBE0 functional mixes the Perdew–Burke–Ernzerhof (PBE) exchange energy and Hartree–Fock exchange energy in a 3:1 ratio, along with the full PBE correlation energy [88,89]. In our earlier studies on ZnO and ZnO/ZnS chemical systems, hybrid functionals had shown the best accuracy when computing the structural features [84,90,91,92]. We note that repeating the calculations using several different ab initio methods is particularly useful to get some feeling for the quantitative validity of the results [93,94,95]. For the crystallographic analysis and visualization, we used KPLOT [96] and the VESTA [97] software.

#### 2.2.2. Electronic Band Structure Calculations

Each ab initio calculation utilizes an all-electron basis set (AEBS) based on Gaussian-type orbitals (GTO) [98]. In the case of *Zn^2+^*, a [*6s5p2d*] basis set was employed as in Refs. [90,99,100]. For *O^2^*^−^, a [*4s3p*] basis set was used as in Refs. [90,101,102]. For *S^2^*^−^, an [*5s4p1d*] all-electron basis set was utilized as in Refs. [103,104], while their combination was applied as in Refs. [40,84]. A *k*-point sampling net of size 8 × 8 × 8 was used. The labels of the special points of the Brillouin zones of the calculated band structures in the case of the idealized wurtzite modification correspond to those of a hexagonal *hcp* lattice, in the case of the fully relaxed distorted wurtzite structure to those of a monoclinic lattice, while in the case of sphalerite the zones correspond to those of a cubic *fcc* lattice. For the visualization of the Brillouin zones, the Bilbao Crystallographic Server [105] and KVEC [106,107] databases were used, while the Xmgrace program [108] was employed for the visualization of band structures.

## 3. Results

### 3.1. XRD Analysis and Rietveld Refinement of ZnO/ZnS Core–Shell Nanoparticles

**Figure 1:**
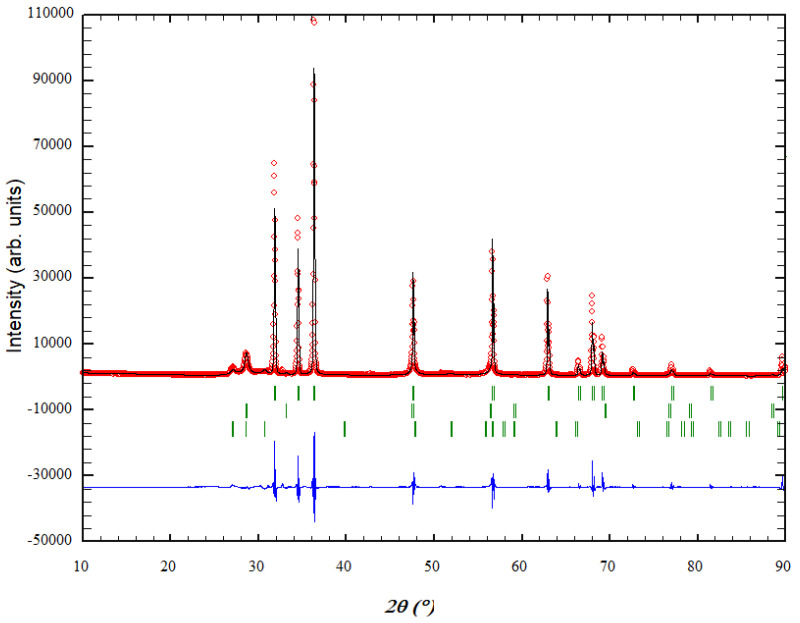
Rietveld diagram of ZnO/ZnS powders obtained after heating at 400 °C for 6 h. The blue line denotes the difference between the experimental (red diamonds) and theoretical (black line) profile, while the Bragg positions are indicated by vertical green slashes. The first, second, and third rows of green bars correspond to the diffraction lines of the ZnO wurtzite (2H) phase, the ZnS sphalerite (3C) phase, and the ZnS wurtzite (2H) phase, respectively.

The X-ray analysis showed a three-phase composition for the investigated sample, where the ZnO phase is dominant (ca. 83.3 (6) mass%) and, interestingly, the auxiliary ZnS compound occurs in two phases (ca. 16.7 (3) mass%) (Figure 1). The dominant ZnO phase appears in the wurtzite (2H) structure (space group P6_3_mc, no. 186), with unit cell parameters a = 3.24801(3) Å and c = 5.20265(8) Å, where the zinc atom is located on the 2b Wyckoff position with coordinates (0.33333 0.66667 0.0000), and the oxygen atom on the 2b position (0.333333 0.666667 0.38202). The second (i.e., first ZnS) phase (ca. 10.2 (2) mass%) appears in the sphalerite (or zinc blende, 3C) structure (space group F-43m, no. 216), with unit cell parameter a = 5.4121(8) Å; the zinc atom is located at (0 0 0), while the sulfur atom is at (0.25 0.25 0.25). The third (i.e., second ZnS) phase (ca. 6.5 (3) mass%) appears in the wurtzite (2H) structure (space group P6_3_mc, no. 186), with unit cell parameters a = 3.804(1) Å and c = 6.255(9) Å; here, the zinc atom is located on the 2b Wyckoff position with coordinates (0.33333 0.66667 0.0000), and the sulfur atom on the 2b position (0.333333 0.666667 0.37500). This data is in good agreement with previous observations on related systems [20,21,24,109,110]. This powder was further analyzed by TEM.

### 3.2. Imaging and Analytical TEM Analysis of the Core–Shell Sample

**Figure 2:**
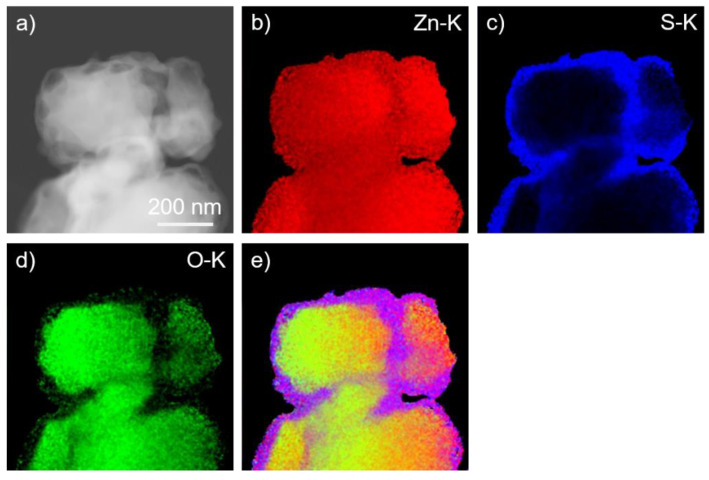
*(***a**) HAADF-STEM image of ZnO/ZnS core–shell nanoparticles with corresponding Zn–K, (**b**) S–K, (**c**) O–K, (**d**) EDX elemental maps, and map (**e**) with superimposed (**b***–***d**) elemental maps.

**Figure 3:**
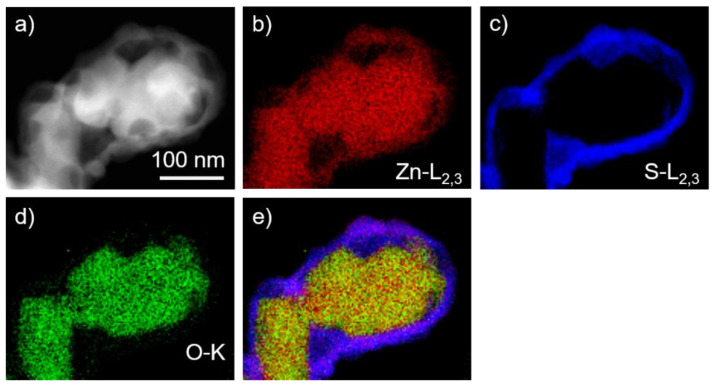
(**a**) HAADF-STEM image of ZnO/ZnS core–shell nanoparticles with corresponding Zn–L_2,3_, (**b**) S–L_2,3_, (**c**) O–K, (**d**) EELS spectrum images, and map (**e**) with superimposed (**b**–**d**) elemental maps.

**Figure 4:**
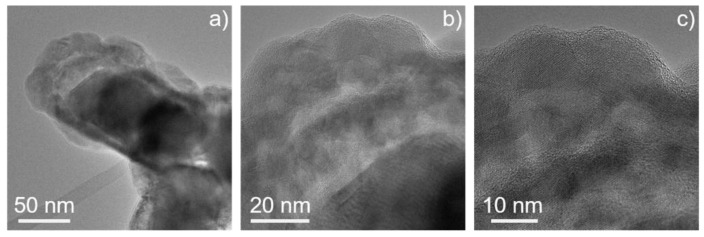
(**a**) Lower magnification and (**b**,**c**) higher magnification HRTEM images acquired from ZnO/ZnS core–shell nanoparticles showing crystalline structure.

We have performed imaging and analytical (S)TEM investigations on the ZnO/ZnS core–shell nanoparticles. EDX elemental mapping using Zn–K, S–K, and O–K peaks shows a homogeneous distribution of the zinc content throughout the particles (Figure 2). The presence of sulfur is limited to the shell part of the particles, while oxygen is detected in the central part. Thus, the particles exhibit a core–shell structure, where ZnS is in the shell and ZnO represents the core of the particles. Quantitative EDX measurements of the ZnS shell confirmed the nominal composition. EELS spectrum imaging (SI) experiments (Figure 3) performed in the energy range of Zn–L_2,3_, S–L_2,3,_ and O–K edges confirmed the presence of a ZnS shell that is formed around the ZnO core. HRTEM images of such ZnO/ZnS core–shell nanoparticles are presented in Figure 4. Our results are in good agreement with earlier experimental observations [110,111,112].

Comparing the spatial distributions of S and O atoms by visual inspection suggests that the interface between the ZnO phase and the regions containing the two ZnS phases is rather thick (several nm), which might indicate the possible existence of some ZnO_1−x_S_x_ polytype, although their volume would be below the detection limit of the XRD. The sulfidation process of ZnO leads to the growth of a ZnS layer on the outside of the ZnO core particles. This growth involves the diffusion of oxygen through the developing ZnS layer from the ZnO–ZnS internal interface to the ZnS surface, as has been suggested in the past [113].

### 3.3. Ab Initio Modeling and Structure Analysis

As a first step, we modeled pristine ZnO and ZnS phases at the ab initio level using hybrid PBE0 and HSE06 density functional calculations and compared them to the present phases observed in the core–shell particles. The XRD results of the cell parameters for the dominant zinc oxide phase in the wurtzite (2H) structure are a = 3.24801(3) Å and c = 5.20265(8)Å, while the PBE0 calculations show a = 3.26 Å and c = 5.20 Å, and the HSE06 ones show a = 3.27 Å and c = 5.20 Å. For the ZnS phase in the sphalerite (3C) structure, the experimental unit cell parameter a = 5.4121(8) Å is in good agreement with PBE0 and HSE06 calculations, showing a = 5.45 Å and a = 5.46 Å, respectively. In the case of the ZnS phase with the wurtzite (2H) structure, we obtain a = 3.804(1) Å and c = 6.255(9) Å from our XRD measurements, while PBE0 calculations show a = 3.86 Å and c = 6.28 Å, and HSE06 calculations show a = 3.86 Å and c = 6.29 Å.

**Figure 5:**
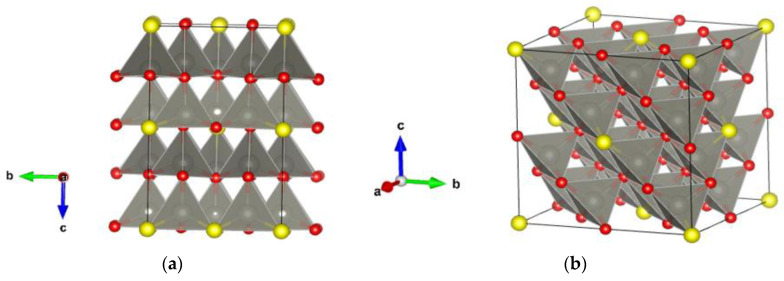
ZnO/ZnS supercell models of (**a**) wurtzite structure with composition ZnO_0.813_S_0.187_; (**b**) sphalerite structure with composition ZnO_0.875_S_0.125_. Grey, yellow and red spheres correspond to zinc, sulfur, and oxygen atoms, respectively.

Furthermore, we have investigated mixed ZnO/ZnS modifications to serve as possible models for the whole core/shell structure or only its interface. A supercell (2 × 2 × 2) model structure of wurtzite and sphalerite was created, and the sulfur/oxygen ratio was set in both supercell models to mimic experimental core–shell structures. In the case of wurtzite, the resulting structure was composed of 81.25% wurtzite ZnO and 18.75% wurtzite ZnS (Figure 5a). The second supercell model was set at 87.5% sphalerite ZnO and 12.5% sphalerite ZnS (Figure 5b). The proposed models are in good agreement with experimental observations from XRD where the ZnO phase is dominant (ca. 83.3 (6) mass%). However, the auxiliary ZnS compound occurs in two phases (ca. 16.7 (3) mass%). Table 1 shows the structural data of proposed supercell models calculated using hybrid PBE0 and HSE06 functionals. In addition, it is possible to model such chemical compositions and nanostructures by using various polytypes, e.g., 5H, 8H, and 15R (more details can be found elsewhere [26,40,84,91]). As in our previous work, we have conducted an additional set of calculations to further analyze and compare our calculated structures with experimentally observed core–shell structures by fixing the atoms to reside on the idealized (high-symmetry) positions in the perfect structure, and only the unit cell parameters were allowed to relax, keeping the space group symmetry (marked as “cell” in Table 1, more details can be found elsewhere [40]).

### 3.4. Band-Gap Measurements of Core–Shell ZnO/ZnS (Nano)particles

**Figure 6:**
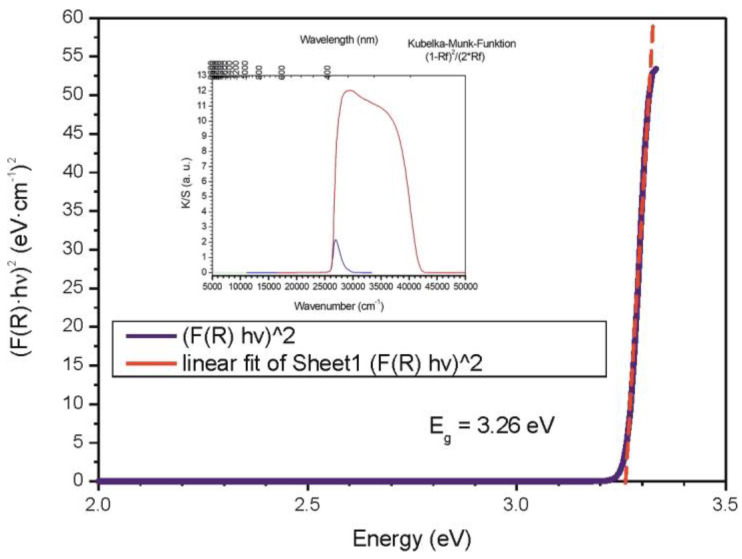
Tauc plots vs. photon energy E for direct band-gap (E_g_) transition together with linear extrapolation. The figure shows the measured size of the band gap of the ZnO/ZnS core/shell structures obtained from diffuse-reflectance spectroscopy (DRS, inset of the figure) of powders.

For the whole spectral range, three different experimental setups (detectors, slits, scan range, and step width) were used. From 280 to 600 nm (UV range), a total of 640 data points were collected by a photomultiplier detector (PMT) with a scan rate of 1.0 nm∙s^−1^, step width of 0.5 nm, and a slit width of 0.1 mm. From 300 to 900 nm (Vis range) and 600 to 2600 nm (NIR range), the total of collected data points were 600 and 500, respectively, with scan rates of 1.0 nm∙s^−1^ and 4.0 nm∙s^−1^ accordingly. For the Vis region, a PMT detector (slit width of 0.06 mm) was used. In the NIR region, the data were collected by a PbS detector at a variable slit width (1.4 to 2.2 mm). In every case, the intensity of BaSO_4_ was measured as a standard I_standard_. The diffuse reflectance F(R) or (K/S) was calculated using the Kubelka–Munk function K/S = ((1 − R_diff_)^2^)/(2R_diff_), where the diffuse reflectance is given by R_diff_ = (I_sample_/I_standard_). From F(R), the Tauc plot can be calculated, which gives the optical band gap of 3.26 eV (Figure 6). The Tauc plot suggests that the ZnO(ZnS) core–shell particles show an allowed direct band-gap transition [114,115,116,117].

### 3.5. First-Principles Band Structure Calculations

A summary of the calculated band gaps for various ZnO/ZnS structures and compositions computed using the PBE0 and HSE06 hybrid functionals on fully relaxed (full) and cell relaxed with high symmetry (cell) structures are shown in Table 2. The best agreement with band-gap measurements of core–shell ZnO/ZnS nanostructures is found when using the PBE0 functional, where the band gap is computed between 3.32 and 3.41 eV for “cell” relaxed structures (with the high symmetry as found in the XRD measurement) compared to the measured band gap of 3.26 eV by diffuse-reflectance spectroscopy (DRS). On the other hand, the results of the HSE06 method on the wurtzite structure show excellent agreement with previous theoretical modeling by Torabi et al., [68] where the band gap of the wurtzite structure—with an approximately similar composition ZnO_0.8_S_0.2_—was computed to be 1.55 eV using HSE06 compared to the present value of 1.53 eV (Table 2). We note that the calculated size of the band gap can vary with the choice of the calculation method, as well as reflect a possible influence of the size of the supercell and the actual positions of the layers of the sulfur atoms in the constructed model structure for a given S:O composition, as observed in our previous study [40].

**Figure 7:**
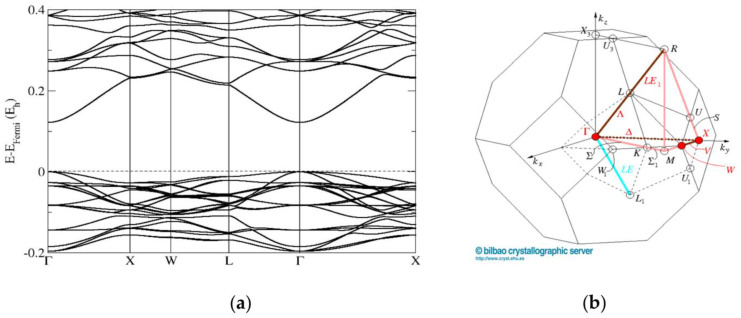
Band structures (**a**) of the sphalerite ZnO_0.875_S_0.125_ model computed using the PBE0 functional with undistorted cubic (F-43m) symmetry and (**b**) corresponding directions in the Brillouin zone (https://www.cryst.ehu.es/cryst/get_kvec.html, accessed on 6 November 2022).

**Figure 8:**
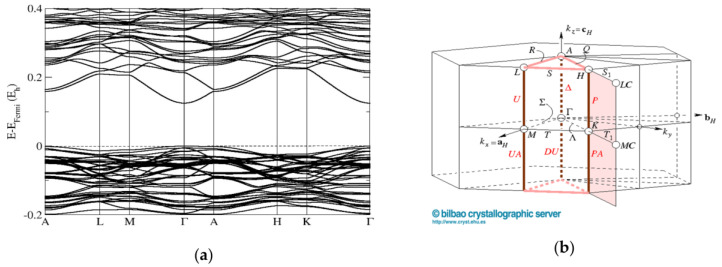
Band structures (**a**) of the wurtzite ZnO_0.813_S_0.187_ supercell model computed using the PBE0 functional with undistorted hexagonal (P6_3_mc) symmetry and (**b**) corresponding directions in the Brillouin zone (https://www.cryst.ehu.es/cryst/get_kvec.html, accessed on 6 November 2022).

The band-structure calculations conducted for the cubic sphalerite ZnO_0.875_S_0.125_ model computed using the PBE0 functional show a direct band gap of 2.76 eV at the Γ point of the Brillouin zone. (Figure 7). The band structure calculations performed for the ZnO/ZnS supercell model with the wurtzite structure (composition ZnO_0.813_S_0.187_) also show a direct band gap of 2.17 eV at the Γ point of the Brillouin zone (Figure 8). These results are in good agreement with our band-gap measurements (Figure 6) from diffuse-reflectance spectroscopy (DRS) of powders, where a direct band gap has been observed.

**Figure 9:**
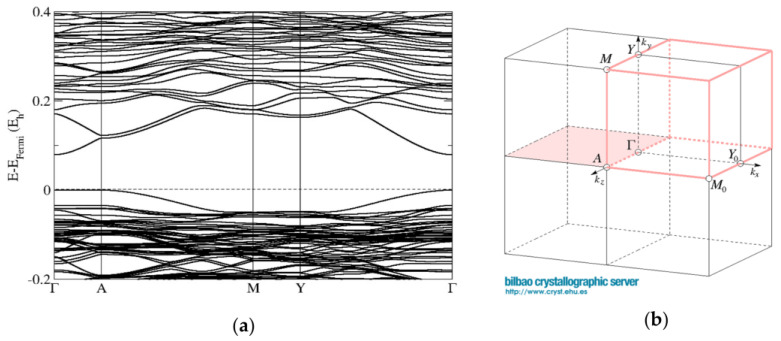
Band structures (**a**) of the wurtzite ZnO_0.813_S_0.187_ supercell model computed using the PBE0 functional with fully relaxed and distorted monoclinic (Cm) symmetry and (**b**) corresponding directions in the Brillouin zone (https://www.cryst.ehu.es/cryst/get_kvec.html, accessed on 6 November 2022).

Moreover, we show the predicted band structures of the wurtzite ZnO_0.813_S_0.187_ supercell model computed using the PBE0 functional with fully relaxed and distorted monoclinic (Cm) symmetry, which, to our knowledge, has not been observed so far (Figure 9). The band structure appears to have a direct band gap; however, we note that the top of the valence band (TVB) at the A point of the Brillouin zone competes with the TVB at the Γ point (Figure 9). A similar effect has been observed previously in various proposed ZnO/ZnS compounds [40]. Furthermore, when computing the polytypic structures of ZnO_0.8_S_0.2_, such as 5H, 8H, and 15R, we have found that it is possible to tune such semiconductor material to semi-metallic and metallic properties, according to DFT calculations using the hybrid approximation (HSE06 and PBE0).

## 4. Discussion

The most common way to obtain mixed ZnO/ZnS compounds is by creating core–shell nanostructures, as in our experiment. The proposed gas-phase sulfidation synthesis method is responsible for the specific structure and composition of the material. Namely, a solid ZnO particle reacts with the surrounding gas (H_2_S), such that inside the solid, two phases are established, an unreacted core and a porous product layer (shell). The process is usually governed by the diffusion of H_2_S through the product layer toward the reaction site, while a gaseous side-product (H_2_O) diffuses from the reaction site inside the particle toward the surface. As a result, there is a sharp boundary between the two solid phases. The (decreasing) core size during the reaction determines the available reaction surface. At the same time, progressive accumulation of the product in the outer layer hinders gas diffusion. Both phenomena affect the reaction kinetics, which becomes very sluggish after a certain period. This synthesis method appears to be very simple and effective, and furthermore, there is no need for special conditions, apparatus, etc.

Our XRD, imaging, and analytical (S)TEM measurements show very good agreement with previous experimental and theoretical results [110,111,112,118,119], where various morphologies have been observed. Lu et al. [59] show that peaks in the diffraction pattern correspond to the wurtzite ZnO and wurtzite ZnS phases. Sharma et al. stated that the cubic sphalerite structure is mostly preferred at room temperature solution synthesis and that the formation of hexagonal ZnS indicates epitaxial growth over ZnO hexagonal wurtzite core particles” [62]. Since in our samples we have both cubic and hexagonal ZnS phases, this suggests that part of the ZnS phase is epitaxially grown on ZnO grains (wurtzite ZnS) and part is independently formed (cubic ZnS). Moreover, in earlier investigations, different phase compositions and the appearance of various polytypic structures have been observed for ZnO/ZnS core/shell systems. Hitkari et al. [58] identified the observed crystal phases as pure wurtzite ZnO and the 8H ZnS polytypic phase. Moraes et al. present a new ZnO/ZnS/carbon xerogel composite, composed of the dominant wurtzite ZnO phase (88%) and minor wurtzite ZnS phase (8.8%), with a small percentage of the new 10H polytype structure also being observed (3.1%) [120]. Kumar et al. report the 15R polytype observed for the first time in ZnS nanowires [121]. While there exist about 200 experimentally identified stacking variants of ZnS [122], ZnO has only three experimentally known bulk phases: wurtzite and sphalerite under ambient conditions and a NaCl phase at high pressures. Since most of the research on mixed ZnO/ZnS compounds and their electronic properties is mainly focused on only two experimentally known phases—wurtzite and sphalerite—finding new polytypic modifications will have a strong impact on the range of accessible electronic and related properties correlated to the structures of the modifications [40,84].

Since the band-gap energies of ZnO and ZnS (3.4 eV and 3.7 eV) are too large for optimal photovoltaic efficiency, they can be controlled by forming heterostructures of ZnO/ZnS [18]. It has been reported previously that strain at the heterostructure interface could reduce the natural band gap slightly [123,124,125,126], whereas only a staggered (so-called type-II) [127,128] band alignment at the interface could give rise to a much smaller band gap than either of the individual core or shell material. The strain and quantum confinement effect are the two reasons which may affect the natural type-II band gap (~1.93 eV) of a ZnO/ZnS core/shell nanowire, and these two effects were considered to predict an effective energy gap at the ZnO/ZnS interface of about 2 eV for very small core/shell nanowires [16,18]. Considering the diameter (150–200 nm) of the ZnO/ZnS core/shell nanowire in these studies, no quantum confinement effect can play a role in modifying the band gap, although a weak effect of strain on the size of the type-II energy gap may exist. The bowing coefficients can be used for describing band-gap reduction. The bowing coefficients increase as the size and chemical mismatch between the constituents increase [124]. For example, the bowing coefficients increase from M^II^S_1−x_O_x_ to M^II^Se_1−x_O_x_ to M^II^Te_1−x_O_x_, because from S to Se to Te, the atomic size difference increases. However, the volume of the interface in our core/shell system appears to be too small—in comparison to the heterostructures discussed above—to expect a noticeable effect in the UV/Vis measurements, even if a staggered type-II band gap were present.

The experimental band-gap measurement in our study coincides with earlier experimental and theoretical findings on core/shell nanostructures. Our core–shell structure is composed of 83.6% ZnO and 16.7% ZnS; thus, if the band-gap measurements were to reflect just the total amount of material, we would expect it to yield a value close to the band gap of the dominant ZnO 2H phase. However, the surface layer consists of ZnS, and therefore the measured band gap could be assumed to reflect the band gap of ZnS instead. However, since the band gap of pristine ZnS is larger than the one for pristine ZnO, radiation below the band-gap energy of ZnS would be expected to cross the relatively thin ZnS shell essentially unimpeded, and thus the observed band gap would again reflect the one of the ZnO core. Nevertheless, the band gap lies below the one of bulk ZnO. This can be understood by noting that one would generally expect that disorder effects, such as various defects associated with the nanometer size of the core–shell particles, would lower the band gap compared to the bulk material, explaining why our measured band gap lies below the experimental band gap for bulk ZnO.

We also note that one could propose the hypothesis that the narrowing of the band gap might be a reflection of the presence of the interface region between the ZnO core and the ZnS shell. As mentioned above, the results of the HSE06 functional calculations (1.53 eV) on the wurtzite structure show excellent agreement with the previous theoretical modeling by Torabi et al. [68], where the band gap of the wurtzite structure with similar composition was computed to be 1.55 eV using HSE06 compared to the value of 1.53 eV we have computed. Our band-gap calculation (PBE0 2.76 eV, HSE06 2.13 eV) for the fully relaxed structures also concurs with the work by Schrier et al. [18], who find a band gap of 2.31 eV for ZnO/ZnS bulk heterostructures consisting of ZnO/ZnS slabs of sphalerite, but with 1:1 ratio of ZnO/ZnS.

However, these fully relaxed structures are theoretical models for an infinite crystal with a unit cell reflecting the composition and local structure of the interface, i.e., if we were to synthesize a mesoscopic crystal of this structure and composition, we would expect to measure a band gap similar to the computed ones. However, as mentioned above, the volume of the interface region, although noticeable in the S(TEM)/EDX/EELS analysis, appears to be too small to dominate the band-gap measurements. Thus, fully relaxed structures do not correspond to the phases seen in the core–shell material for which the experimental measurements have been performed. As a consequence, our earlier argument applies, and the reduction in the band gap of the core–shell particle compared to the bulk ZnO is mostly due to the nanoscopic size of the particles. The best agreement with band-gap measurements of core–shell ZnO/ZnS nanostructures is achieved with the PBE0 functional, where the band gap is computed to lie between 3.32 and 3.41 eV on “cell” relaxed structures (with the same high symmetry as found in the experiment) compared to the measured band gap of 3.26 eV by diffuse-reflectance spectroscopy (DRS). One possible explanation might be that the interface structure is still dominated by the structures of the pristine ZnO and ZnS in the core and shell regions adjacent to the interface, which prevent strong lattice distortions, and thus the interface plus the adjacent regions in the core and the shell together are large enough to display the band gap of a slightly strained compositionally mixed but structurally homogeneous ZnO/ZnS region.

## 5. Conclusions

ZnO/ZnS core/shell nanostructures were synthesized by gas-phase sulfidation of ZnO powder at elevated temperatures, which is fast, simple, and inexpensive compared to other synthesis methods. The structural, morphological, and local element composition of the ZnO/ZnS core/shell nanostructures were characterized by XRD, imaging, and analytical (S)TEM and concur with literature data. The XRD results showed a three-phase composition, where the ZnO phase is dominant and appears as a wurtzite structure, and the ZnS phase appears in both the cubic sphalerite and the hexagonal wurtzite modification. The (S)TEM combined with EDX and EELS further show that the nanomaterials are core/shell structures with a relatively thick interface, where the shell and core consist of ZnS and ZnO, respectively.

In particular, we have studied the electronic properties of the investigated ZnO/ZnS chemical systems, where a band-gap measurement of the ZnO/ZnS core/shell sample has been performed. The optical band gap of 3.26 eV has been measured using a Tauc plot, suggesting that the ZnO/ZnS core/shell particles show an allowed direct band-gap transition, reflecting most likely the band gap of the nanosized ZnO core. Theoretical supercell models were created to investigate the structure and electronic properties of ZnO/ZnS. DFT hybrid calculations were performed using PBE0 and HSE06 functionals. The com puted band structures concur with the UV/Vis DRS measurements, where a direct band gap has been observed. Additional band structures and band-gap tuning depending on the structural features have been suggested.

This theoretical analysis of model systems describing the interface region demonstrates the large range of band-gap values possible in ZnO/ZnS nanostructures. When analyzing the structure–property relationship, we have found two major relevant aspects of such ZnO/ZnS materials: possible structure distortions leading to a lower (monoclinic) symmetry, as well as the relative amounts of sphalerite and/or wurtzite structure modifications in the ZnO/ZnS materials. Both of these features can dramatically reduce the size of the band gap. Together with our experimental results on core/shell ZnO/ZnS nanoparticles, they underline the excellent potential of ZnO/ZnS heterostructures of the core/shell type for band-gap engineering, with promising applications in many areas, such as photocatalysis, semiconductors, photovoltaics (PVs), light-emitting diodes (LEDs), laser diodes, solar cells, infrared detectors, or thermoelectrics.

## Figures and Tables

**Table 1:** Calculated structural data of the ZnO/ZnS supercell models with various ZnO/ZnS compositions. Cell parameters are given in Angstrom (Å). Local optimizations were performed with the PBE0 and HSE06 hybrid functionals.

Structure and Composition	Space Group and Unit Cell Parameters (Å)			
	*HSE06 (Full)*	*HSE06 (Cell)*	*PBE0 (Full)*	*PBE0 (Cell)*
**Sphalerite** **ZnO_0.875_S_0.125_**	*F*-43*m* (no. 216) *a* = 4.689	*F*-43*m* (no. 216) *a* = 4.719	*F*-43*m* (no. 216) *a* = 4.687	*F*-43*m* (no. 216) *a* = 4.717
**Wurtzite** **ZnO_0.813_S_0.187_**	*Cm* (no. 8) *a* = 11.675, *b* = 6.727, *c* = 10.845, *β* = 90.39	*P*6_3_*mc* (no. 186)) *a* = 3.358, *c* = 5.379	*Cm* (no. 8) *a* = 11.663, *b* = 6.724, *c* = 10.839, *β* = 90.39	*P*6_3_*mc* (no. 186)) *a* = 3.407, *c* = 5.451

**Table 2:** Summary of the calculated band gaps for various ZnO/ZnS structures and compositions. Ab initio calculations were carried out using the PBE0 and HSE06 hybrid functionals on fully relaxed (full) and “cell” relaxed with high symmetry (cell) structures.

Structure and Composition	Computed Band Gap Size (eV)			
	*HSE06 (Full)*	*HSE06 (Cell)*	*PBE0 (Full)*	*PBE0 (Cell)*
**Sphalerite** **ZnO_0.875_S_0.125_**	2.13	2.70	2.76	3.32
**Wurtzite** **ZnO_0.813_S_0.187_**	1.53	2.79	2.17	3.41

## Data Availability

Not applicable.
